# Carbon microelectrodes for the measurement of neurotransmitters with fast-scan cyclic voltammetry: methodology and applications

**DOI:** 10.3389/fbioe.2025.1569508

**Published:** 2025-04-07

**Authors:** Nadiah Alyamni, Jandro L. Abot, Alexander G. Zestos

**Affiliations:** ^1^ Department of Biomedical Engineering, The Catholic University of America, Washington, DC, United States; ^2^ Department of Chemistry, American University, Washington, DC, United States; ^3^ Department of Mechanical Engineering, The Catholic University of America, Washington, DC, United States

**Keywords:** biosensor, behavioral responses, carbon electrode, fast-scan cyclic voltammetry (FSCV), neurotransmitter

## Abstract

Carbon microelectrodes (CMEs) have emerged as pivotal tools in the field of neurochemical sensing, enabling precise, real-time monitoring of neurotransmitters in both research and clinical contexts. The current review explores the design, fabrication, and application of CMEs, emphasizing recent advancements in material science and electrochemical techniques that enhance their sensitivity, selectivity, and biocompatibility. Innovations such as the incorporation of nanomaterials, including graphene and carbon nanotubes, and the adoption of advanced fabrication methods like three-dimensional (3D) printing and chemical vapor deposition, are discussed in detail. These developments have led to significant improvements in electrode performance, the reduction of biofouling and interferants, while enabling the detection of low concentrations of neurochemicals in complex biological systems. This review further highlights the potential of CMEs to address clinical challenges such as diagnosing and monitoring neurological disorders such as Parkinson’s Disease and depression. By integrating advanced surface modifications, polymer coatings, and method development strategies, CMEs demonstrate high durability, reduced fouling, and enhanced specificity. Despite these advancements, challenges remain related to long-term *in vivo* stability, batch fabrication, and reproducibility, thus necessitating further research and optimization. This review highlights the transformative potential of CMEs in both research and therapeutic applications, providing a comprehensive overview of their current state and future directions. By addressing existing limitations and leveraging emerging technologies, CMEs have the potential to further enhance neurochemical sensing and contribute to breakthroughs in neuroscience and biomedical science.

## 1 Introduction

Neurotransmitters are essential chemical messengers that facilitate communication between neurons, influencing various physiological functions and playing a significant role in numerous diseases and influence processes such as mood, cognition, and motor control (Teleanu et al., 2022). Based on their molecular structure, neurotransmitters include monoamines, amino acids, acetylcholine, and neuropeptides. Among the monoamines, Dopamine (DA), norepinephrine (NE), epinephrine (EP), and serotonin (5-HT) are heavily studied for their involvement in neurological disorders like Parkinson’s Disease and depression (Ferreira, 2015). Amino Acids based neurotransmitters include glutamate (Glu), gamma-aminobutyric acid (GABA), aspartate (Asp), and tyrosine (Tyr) are involved in excitatory and inhibitory signaling in the central nervous system (Norris et al., 2024). Neuropeptides include neuropeptide Y (NPY) which is involved in regulation of stress and depression (Morales-Medina et al., 2010). Acetylcholine (ACh) is another neurotransmitter that is crucial for muscle activation and memory-related functions (Picciotto et al., 2012). Other neurochemicals include molecules such as nitric oxide (NO), carbon monoxide (CO), and hydrogen sulfide (H_2_S) that also act as gastrointestinal tract signaling agents and influence cellular communication (Farrugia and Szurszewski, 2014).

Given the essential role of neurotransmitters, their precise measurement is vital for advancing disease diagnosis and understanding pathological processes. However, the detection of neurotransmitters in biological samples such as urine, blood, or cerebrospinal fluid poses significant challenges due to their structural similarities and low concentrations (Rejithamol and Beena, 2022). Biosensors have become indispensable tools in biomedical research and clinical applications, providing an interface between biological systems and digital technologies to monitor, measure, and analyze biochemical processes (Kaur et al., 2018). A biosensor typically comprises three essential components: a biological recognition element, a transducer, and a signal processor. The biological recognition element interacts with the target analyte (e.g., enzymes, antibodies, or nucleic acids), while the transducer converts this interaction into a quantifiable electrical signal, which is then processed and displayed for interpretation (Pohanka and Skládal, 2008).

When choosing appropriate biosensors for neurotransmitter detection, researchers must evaluate several critical performance parameters. The sensitivity and detection limits must align with physiological neurotransmitter concentrations found in blood serum (Ozel et al., 2015). A major challenge is achieving sufficient selectivity, as biological samples contain numerous potentially interfering compounds such as ascorbic acid or uric acid to name a few (Jackowska and Krysinski, 2013). Moreover, the reliability of measurements depends heavily on sensor reproducibility and resistance to biofouling - a phenomenon where proteins and other biomolecules accumulate on electrode surfaces, compromising sensor function and diminishing conductivity by blocking biomolecule adsorption on the microelectrode (Hanssen et al., 2016). Developing fouling-resistant surfaces remains an active area of research in biosensor design. The simultaneous detection of multiple neurotransmitters presents additional complexity due to their structural and chemical similarities (Si and Song, 2018). While current detection methods have limitations, emerging neurotechnology shows promise in enabling high-throughput, selective monitoring of neural signaling molecules with minimal tissue disruption (Luan et al., 2023).

Among the diverse array of biosensors, carbon microelectrodes (CMEs) have gained prominence for their role in neurochemical sensing. CMEs possess unique properties such as high biocompatibility, and exceptional spatiotemporal resolution, making them ideal for monitoring fast and dynamic biochemical changes at the cellular level (Devi et al., 2021). While some CMEs share similar size ranges with metal microelectrodes, carbon fiber electrodes stand out due to their ultrasmall dimensions with minimal tissue damage has made them as essential tools for neuroscience research (Bucher and Wightman, 2015). The detection of neurotransmitters such as dopamine, serotonin, and glutamate is vital for understanding the pathophysiology of neurological disorders like Parkinson’s Disease, depression, and epilepsy (Venton and Wightman, 2003).

Recent advancements have focused on improving CMEs by integrating nanomaterials such as carbon nanotubes (CNTs), graphene, and metal oxide nanoparticles. These modifications enhance their electrical conductivity, durability, sensitivity and selectivity, thus enabling lower limits of detection and reducing surface fouling and background noise. For instance, CNTs increase the surface area and electron transfer rates of CMEs, significantly improving their sensitivity to electroactive molecules through enhanced conductivity and increased surface area and roughness (Jain et al., 2017). These enhancements have expanded the applications of CMEs in real-time, high-resolution monitoring of neurotransmitter dynamics during complex behaviors and therapeutic interventions (Mendoza et al., 2020).

In addition to neurochemical sensing, CMEs have demonstrated utility in broader biomedical applications, such as monitoring oxidative stress markers, detecting metabolite biomarkers, and investigating drug interactions in the body and brain (Puthongkham and Venton, 2020). Their integration with emerging technologies such as microfluidics and wearable devices promises to revolutionize personalized medicine by enabling continuous, non-invasive monitoring of biochemical signals. This review highlights the latest advancements in CME-based biosensors, exploring their fabrication, functionalization, and applications in diagnosing and treating neurological disorders. It emphasizes their pivotal role in unraveling the complex interplay of neurochemical processes and behaviors, paving the way for innovative clinical and research methodologies.

## 2 Fabrication of CMEs

### 2.1 Carbon fiber microelectrodes (CFMEs)

**FIGURE 1:**
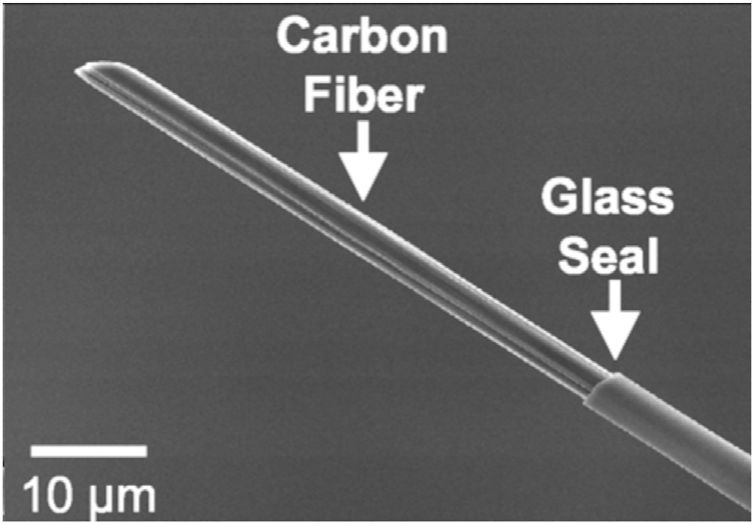
Scanning electron micrograph (SEM) image of a carbon fiber aspirated into a glass capillary and pulled with a capillary puller to form a CFME. Figure is reproduced with permission (Robinson et al., 2003).

Due to their excellent physicochemical and electrochemical properties, carbon fibers are commonly used electrode materials. The electrical (electrochemical), thermal, density, and elastic modulus properties of CMEs are excellent and conducive towards biosensing. The microscale diameter (∼7–10 microns) of carbon fibers makes them optimal to be used as microelectrodes. Carbon fiber microelectrodes (CFMEs) are constructed when carbon fibers are aspirated and insulated in pulled glass capillary and other related insulations. CFMEs have been characterized with scanning electron microscopy (SEM) imaging to properly observe their microstructure and surface features (Figure 1) (Robinson et al., 2003). CFMEs have been used as high-performance biosensors to detect neurotransmitters such as dopamine (DA) to monitor signal production in single cells *in vivo* (Lu et al., 2017).

Carbon fibers can be derived from different precursors, primarily polyacrylonitrile (PAN) and pitch-based sources. The choice of precursor material significantly affects the electrochemical properties of the resulting carbon fibers. PAN-based fibers, such as T-650 carbon fibers are known for their high tensile strength and elastic modulus. These fibers exhibit faster electron transfer kinetics, which is beneficial for the rapid detection of neurotransmitters. Moreover, they also have relatively low capacitance and background charging currents, which can improve the signal-to-noise ratio in electrochemical measurements (Huffman and Venton, 2008). Beyond differences in carbon fiber conductivity, the exposed surface area of CFMEs also significantly influences their capacitive charge/discharge response. A larger surface area can enhance charge storage capacity, increase background currents, and improve analyte adsorption, thereby affecting overall sensitivity and electrochemical performance in FSCV applications (Castagnola et al., 2020; Rafi and Zestos, 2021).

On the other hand, pitch-based fibers, such as Cytec Thornel ^®^ P-55, are derived from petroleum or coal tar pitch and are characterized by higher conductivity compared to PAN-based fibers. These fibers can handle larger currents, which is advantageous when detecting analytes that produce larger oxidation currents, such as ascorbic acid and 3,4-dihydroxyphenylacetic acid (DOPAC). Pitch-based fibers, however, tend to have higher background charging currents, which can sometimes hinder the interpretation of electrochemical signals (Huffman and Venton, 2008). Electrochemical treatments and surface modifications can enhance the sensitivity and selectivity of carbon fiber microelectrodes. For instance, electrochemical treatments in KOH can increase porosity and regenerate the carbon surface and introduce more oxygen functional groups, which are beneficial for adsorption and electron transfer (Cao et al., 2021).

#### 2.1.1 Implications and current advancements for CFME

Iost et al. measured laboratory rats' glucose levels with a CFME biosensor. Glucose oxidase enzyme was electrodeposited onto the electrode surface to metabolize glucose to hydrogen peroxide, which was then oxidized and measured with voltammetry at the electrode surface. Electrochemical analysis and environmental pollution monitoring are two other popular CFME uses. These electrodes have a thin diffusion layer, internal resistance (IR) drop, renewable surface and good signal-to-noise ratio (Iost et al., 2015). The internal resistance (IR) drop, also known as the ohmic drop, refers to the voltage loss that occurs due to the inherent resistance of the electrolyte, electrode material, and electrical connections within the electrochemical system (Oelßner et al., 2006). This resistance impedes efficient charge transfer and can lead to distortions in the recorded electrochemical signal, particularly at high scan rates where fast electron transfer kinetics are required. With FSCV, IR drop can influence peak potentials and broadening of voltammetric responses, ultimately affecting sensitivity and resolution (Holmes, 2019).

A related study reported that using innovative carbon nanomaterial electrodes can enhance neurochemical detection (Mendoza et al., 2020). These advanced carbon nanomaterials may circumvent CFME limitations such as sensitivity, temporal resolution, and fouling. Researchers hope to increase neurochemical sensing and overcome CFME limitations by utilizing high surface area, superior conductivity, and biocompatibility of these nanomaterials (Mendoza et al., 2020). CFMEs possess several advantageous properties. Their electrode surface can be etched and renewed, they can be functionalized with negatively charged oxide groups to enhance sensitivity, and they exhibit excellent biocompatibility and strong efficacy for monoamine adsorption. Some studies investigated dopamine adsorption at surface-modified carbon-fiber electrodes, demonstrating that surface modifications can enhance sensitivity and specificity for dopamine detection (Bath et al., 2001). They also showed that dopamine could adsorb to and desorb from CFMEs within sub-second timeframes, providing high temporal resolution for neurochemical monitoring (Bath et al., 2000).

Moreover, others developed assays for studying carbon microelectrodes with renewable surfaces, allowing for prolonged use and consistent performance in detecting neurotransmitters (Takmakov et al., 2010) when scanning to potentials such as 1.3 V. It was found that the overoxidation of CFMEs enhances dopamine adsorption and enhancing sensitivity, making these electrodes highly effective for *in vivo* measurements (Heien et al., 2003). Specific oxygen-containing functional groups were identified on the carbon surface that underlie enhanced sensitivity to dopamine, further improving the performance of CFMEs (Roberts et al., 2010).

Combining CFMEs with fast-scan cyclic voltammetry (FSCV) has emerged as a powerful technique for neurotransmitter detection. This approach allows researchers to monitor and analyze neurotransmitter dynamics with high spatiotemporal resolution and sensitivity enabling the detection of fast neurotransmitter dynamics. The relatively small CFME (∼7 microns) diameter allows for sampling in small subregions of the brain that can be easily differentiated from one another and identified via histology. The fast scanning rate of the voltage waveform (typically in the range of 100–1,000 V/s) allows for the measurement of neurotransmitter release and reuptake on a sub-second timescale comparable to the phasic firing of dopaminergic neurons (Zestos, 2018). This capability is particularly useful for studying rapid neurotransmission events and their correlation with behavior. Furthermore, CFMEs utilized with FSCV have been combined with optogenetic or electrical stimulation techniques to probe the causal relationship between neurotransmitter release and behavior in neurons expressing channel rhodopsin. By precisely controlling the timing and location of stimulation, researchers can investigate how specific neurotransmitters modulate neural circuits and contribute to behavioral responses (Diesner, 2018).

While CFMEs provide high spatial resolution, their temporal resolution is inherently limited by a combination of material properties and the applied detection technique. The charge transfer kinetics, adsorption/desorption rates of analytes, and background charging currents associated with the carbon fiber surface influence the effective temporal resolution (Yang et al., 2017). Additionally, with FSCV, the frequency of waveform application determines the rate at which measurements are taken. Faster waveform scanning improves temporal resolution (Roberts and Sombers, 2017). Conversely, lower scan frequencies reduce noise but compromise the ability to detect rapid neurotransmitter fluctuations. Therefore, optimizing both the electrode material and the FSCV waveform parameters is critical for achieving high neurochemical detection with minimal trade-offs.

Recent advances in CFME array technology have enabled the development of high-density, minimally invasive electrode arrays for chronic *in vivo* applications. These advancements facilitate simultaneous multisite neurochemical sensing and electrophysiological recording, improving spatial resolution while minimizing tissue disruption. Scalable fabrication techniques, such as photolithography and flexible electrode integration, allow for reproducible and high-density CFME arrays that enhance neurochemical monitoring in deep brain structures (Banerjee et al., 2020). Scientists have developed high-density carbon fiber arrays capable of chronic electrophysiology and fast-scan cyclic voltammetry (FSCV) for neurotransmitter sensing (Patel et al., 2020). These arrays enable multimodal measurements with minimal tissue damage and high temporal resolution. Schwerdt et al. (2017) designed subcellular-scale electrode arrays with multiple carbon fiber recording sites, demonstrating the ability to map dopamine release at multiple locations in deep brain structures. Xia et al. (2023) introduced a scalable, flexible carbon fiber electrode thread array, which allows for three-dimensional neurochemical probing in deep brain regions, advancing the capability of CFME arrays for high throughput measurements. One study proposed a glassy carbon fiber-like multielectrode array (GCF-MEA) fabricated via photolithography, allowing batch production of highly reproducible CFME arrays for chronic neurochemical sensing (Castagnola et al., 2024).

### 2.2 Polymer modified carbon electrodes

Polymer-modified carbon electrodes have demonstrated significant potential in enhancing the sensitivity, selectivity, and biocompatibility of neurotransmitter detection, particularly in complex biological environments. Conducting polymers such as poly (3,4-ethylenedioxythiophene) (PEDOT), Nafion, and polyethylene glycol (PEG) are commonly incorporated onto carbon fiber microelectrodes to improve charge transfer, reduce immune responses, and promote tissue integration (Patel et al., 2016). For instance, PEDOT: Nafion coatings have been shown to enhance dopamine detection in the zebrafish retina through timed electrodeposition, underscoring their utility for precise neurotransmitter measurements (Cho et al., 2020). Similarly, PEDOT-polystyrene sulfonate (PEDOT-PSS) coatings enhance long-term electrode stability by reducing electrical resistance and improving charge transfer capacity (Vomero et al., 2017). However, while PEDOT/pTS coatings have proven beneficial for chronic electrophysiology applications, their high surface area and low impedance may not be suitable for fast-scan cyclic voltammetry (FSCV) due to capacitive charging currents (Taylor et al., 2017). Additionally, although PEDOT/GO coatings can enhance dopamine sensitivity *in vivo*, excessive thickness can lead to increased impedance and hinder FSCV applications.

Similarly, Vreeland and colleagues reported that PEDOT: Nafion composite electrode coatings are biocompatible and capable of selective *in vivo* neurotransmitter detection, underscoring their practical applicability in neurochemical research (Vreeland et al., 2015). Furthermore, other studies have highlighted that polymer modifications on carbon fiber microelectrodes, along with waveform alterations, enhance the detection of neurotransmitter metabolites, thus broadening the utility of these sensors in various analytical settings (Raju et al., 2019). Moreover, further studies reinforced these findings, demonstrating that polymer-modified carbon fiber microelectrodes were capable of precise measurements of neurotransmitter metabolites that are crucial for understanding complex neurochemical interactions (Wonnenberg et al., 2020).

Additionally, other work has compared different polymer nanocomposites, where carbon nanotubes (CNTs) were dispersed within Nafion and overoxidized polypyrrole matrices. These polymeric nanocomposites significantly enhanced the detection of neurotransmitters such as adenosine, further supporting the effectiveness of CNT-polymer nanocomposites in electrochemical sensors (Peairs et al., 2011; Ross and Venton, 2012). These studies collectively highlight the advancements in polymer-modified electrodes, paving the way for more accurate and reliable neurotransmitter measurements during complex behaviors.

Although surface modifications such as polymer coatings or conductive nanomaterials can enhance analyte adsorption and electron transfer, they also increase the electrode’s effective surface area (Cao et al., 2019). With FSCV, this can lead to elevated capacitive charging currents and higher background signals, which may obscure neurotransmitter detection and reduce signal-to-noise ratios (Delmo et al., 2023). Careful optimization of electrode surface treatments is necessary to balance sensitivity improvements with maintaining stable and reproducible background currents.

### 2.3 Carbon nanotubes (CNTs)

Carbon nanotubes (CNTs) are cylindrical nanostructures composed of carbon atoms arranged in a hexagonal lattice. They exhibit extraordinary electrical, mechanical, and thermal properties due to their unique structure, where each carbon atom is bonded to three other carbon atoms, forming a seamless, hollow tube. These properties make CNTs highly conductive and are suitable for use as electrochemical sensors (Iijima, 1991; Iijima and Ichihashi, 1993). CNTs are conductive because they contain sp^2^ hybridized carbon atoms, which allow for the free movement of delocalized electrons along the tube. The high surface area of CNTs also enhances their sensitivity, allowing for the detection of relatively low quantities of analytes, including neurotransmitters (Jacobs et al., 2010).

CNTs have been used to modify carbon fiber microelectrodes (CFMEs), significantly enhancing their performance. The modification of CFMEs with CNTs increases the electrode surface area and promotes electron transfer, resulting in higher sensitivity and lower detection limits for neurotransmitters. Functional groups on CNTs can further modulate the sensitivity and electron transfer kinetics of these electrodes (Jacobs et al., 2011). CNTs have been proven to be efficacious for electrode materials for neurochemical sensing because of their fast electron transfer kinetics due to the ultra-conductive properties of CNTs, high spatiotemporal resolution with high aspect (surface area: volume) ratios that are efficacious for biomolecule adsorption and oxidation, and exhibit a resistance to surface fouling or the formation of non-conductive polymers at the surface that could hinder and prevent biomolecule adsorption.

In addition to modifying CFMEs, CNTs and other carbon nanomaterials have been used to enhance the performance of metal microelectrodes. Another study demonstrated the growth of carbon nanospikes on metal microelectrode sensitivity and the reduction of fouling during dopamine detection (Zestos et al., 2015). Many biomolecules can foul the electrode surface by forming non-conductive polymers that have been shown to coat the electrode surface. Moreover, others demonstrated the growth of carbon nanotubes on metal microelectrodes, which resulted in improved detection of dopamine due to enhanced surface area and electron transfer rates (Yang et al., 2016a). CNT yarns, another type of CNT electrode materials, are created by chemical vapor deposition (CVD) and spinning from vertically aligned CNTs. CNT yarns can also be produced by twisting them together, using a dry-spinning technique borrowed from the textile industry. This approach offers benefits such as scalability, manageability, and the flexibility to modify yarn qualities (Zestos and Venton, 2017). The development of these electrodes provide for novel electrode technology for sensing applications. Also, a floating catalyst CVD method can be employed (Shukrullah et al., 2019) for the fabrication of the CNTs.

Alternatively, wet spinning can be used to form CNT yarn microelectrodes, where a fluid containing nanotubes is extruded to form fibers (Mukai et al., 2016). Moreover, Yang et al. (2017) evaluated different surface properties of CNT yarn and their correlation with electrochemical performance. They concluded that high conductivity, abundant oxygen functional groups, and moderate surface roughness are crucial for high sensitivity and fast electron transfer kinetics (Yang et al., 2017). Another study developed acid-wet spun CNT fiber microelectrodes, which provided even higher sensitivities and faster electron transfer kinetics compared to polyethyleneimine (PEI)-CNT fibers. These fibers maintained sensitivity at high frequencies, allowing for a 2 m sampling rate, which is significantly faster than traditional FSCV (Zestos and Venton, 2017). Further studies explored how different functional groups on CNTs affect the sensitivity and electron transfer kinetics of neurotransmitter detection. They found that carboxylic acid and amide functionalized CNTs significantly increased the oxidative current for serotonin and dopamine, while also enhancing electron transfer rates due to enhancing adsorption of biomolecules on the electrode surface through increased electrostatic interactions between the analytes and the electrode (Jacobs et al., 2011).

CNT yarns produced through these methods exhibit exceptional mechanical strength, electrical conductivity, and thermal stability, making them suitable for biosensing applications (Igbokwe et al., 2019). To further enhance their properties for specific uses, CNT yarns can be functionalized with various polymers. For instance, one study explored CNT fibers functionalized with polyethyleneimine (PEI), which demonstrated higher conductivity and lower overpotentials compared to polyvinyl alcohol (PVA)-CNT fiber microelectrodes. These PEI-functionalized CNT fiber microelectrodes achieved a limit of detection of 5 nM for dopamine, maintained stability for over 10 h, and showed resistance to fouling by serotonin and its metabolite 5-hydroxyindoleacetic acid (5-HIAA) (Zestos et al., 2014).

**FIGURE 2:**
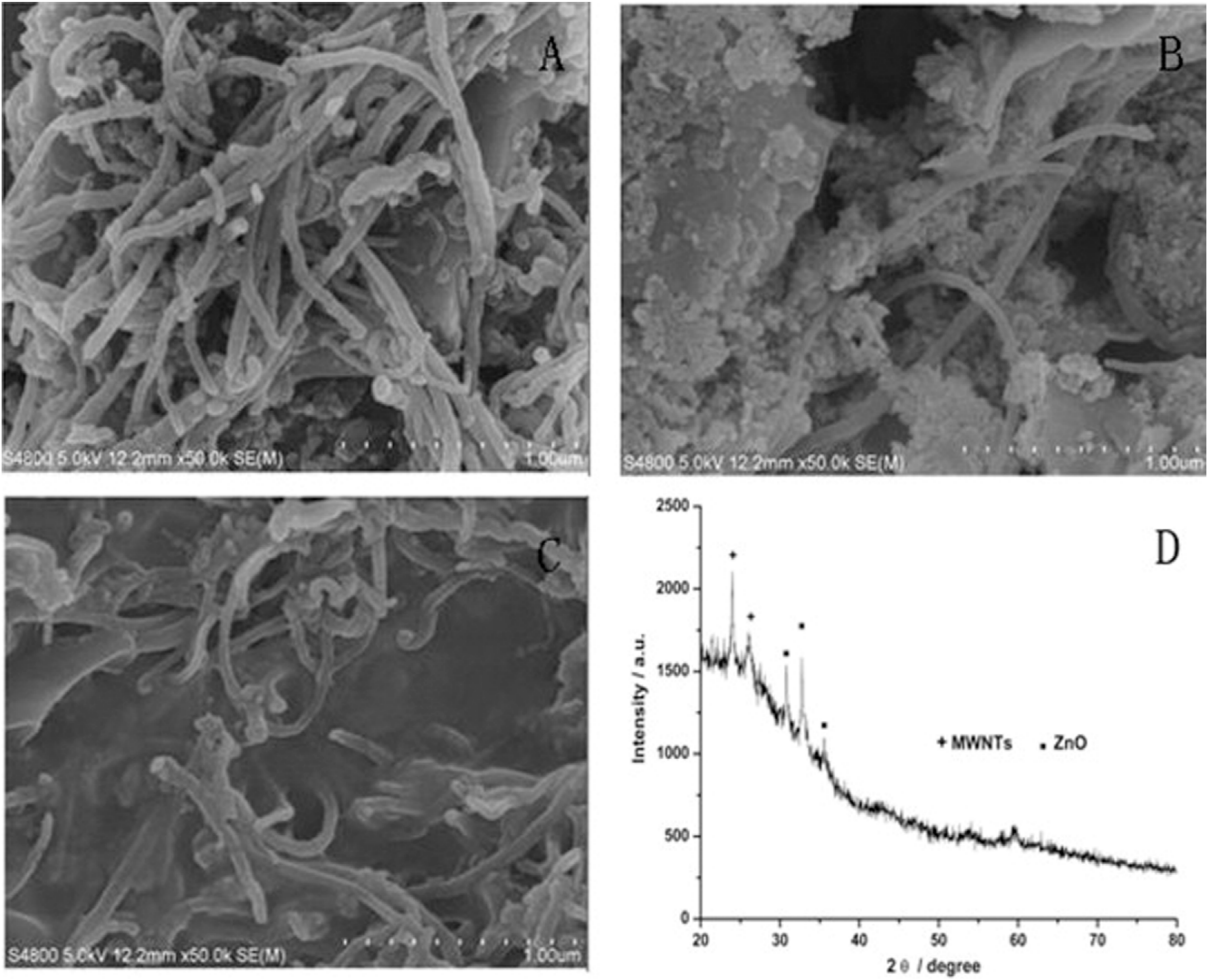
SEM micrographs of **(A)** MWCNTs modified with SPE; **(B)** MWCNTs -ZnO modified with SPE; **(C)** MWCNTs -ZnO/chitosan modified with SPE and **(D)** XRD patterns of MWCNTs -ZnO (Wang et al., 2015).

In another study, researchers developed a sensor based on multi-walled carbon nanotubes (MWCNTs) and ZnO/chitosan composites. This sensor exhibited high catalytic activity and sensitivity for the simultaneous detection of norepinephrine and serotonin in rat cerebrospinal fluid, with detection limits of 100 nM for serotonin (Wang et al., 2015). Figure 2 Illustrates the SEM images of various MWCNTs. The authors found that MWCNTs) coated on screen-printed electrodes (SPE) enhance electron transfer, while the addition of ZnO nanoparticles further improves transmission performance; however, only when chitosan was incorporated did the MWCNTs-ZnO composite form a stable, firmly attached film suitable for simultaneous detection of NE and 5-HT (Wang et al., 2015).

In comparison to CFMEs, CNT-yarn microelectrodes excel in several key areas, including sensitivity and electron transfer kinetics. The improved performance of the CNT-yarn microelectrode is further shown by the fact that the peak separation (Δ_EP_) values are significantly smaller (Mendoza et al., 2020). Recent studies have utilized CNT yarns to fabricate microelectrodes that showed high sensitivity and fast electron transfer kinetics for neurotransmitter detection. These electrodes also resisted surface fouling, making them suitable for long-term *in vivo* measurements (Mendoza et al., 2020). Other researchers have developed microelectrodes with aligned CNT forests, which provided high sensitivity and increased temporal resolution for neurotransmitter detection. This configuration allowed for a nine-fold increase in temporal resolution without a decrease in sensitivity (Xiao and Venton, 2012).

#### 2.3.1 Implications and advancements in CNTs

Various studies have reported that CNT-yarn microelectrodes can improve sensitivity, kinetics, fouling resistance, and stability in neurochemical sensing applications (Yang and Venton, 2017). CNT yarn electrodes, when coupled with FSCV for rapid neurotransmitter detection, exhibit a remarkable feature: their currents for dopamine remain unaffected by the frequency at which the waveform is applied (Yang et al., 2016b). This phenomenon was described because CNT yarn microelectrodes have increased surface roughness including cavity-like structures that have been shown to “trap” biomolecules at the surface, which hindered desorption, therefore, not requiring preconcentration at the negative holding potential. This characteristic holds great potential for enhancing the temporal resolution of neurochemical experiments. Moreover, treatments applied to these yarns, such as laser etching and anti-static gun application, can modulate the surface roughness of the electrodes (Yang et al., 2017c). These modifications enable transient trapping of the analyte, further facilitating the accurate measurement of neurotransmitters. By leveraging the unique properties of CNT yarn electrodes, researchers can not only achieve high temporal resolution in neurochemical experiments, but also manipulate the electrode surface to enhance analyte trapping capabilities, opening up new avenues for advancing our understanding of neurochemical dynamics during various behavioral processes (Yang et al., 2016b; Yang et al., 2017c). The use of nanomaterials for electrode tools with high spatiotemporal resolution will allow for the monitoring of fast and spontaneous complex behaviors.

**FIGURE 3:**
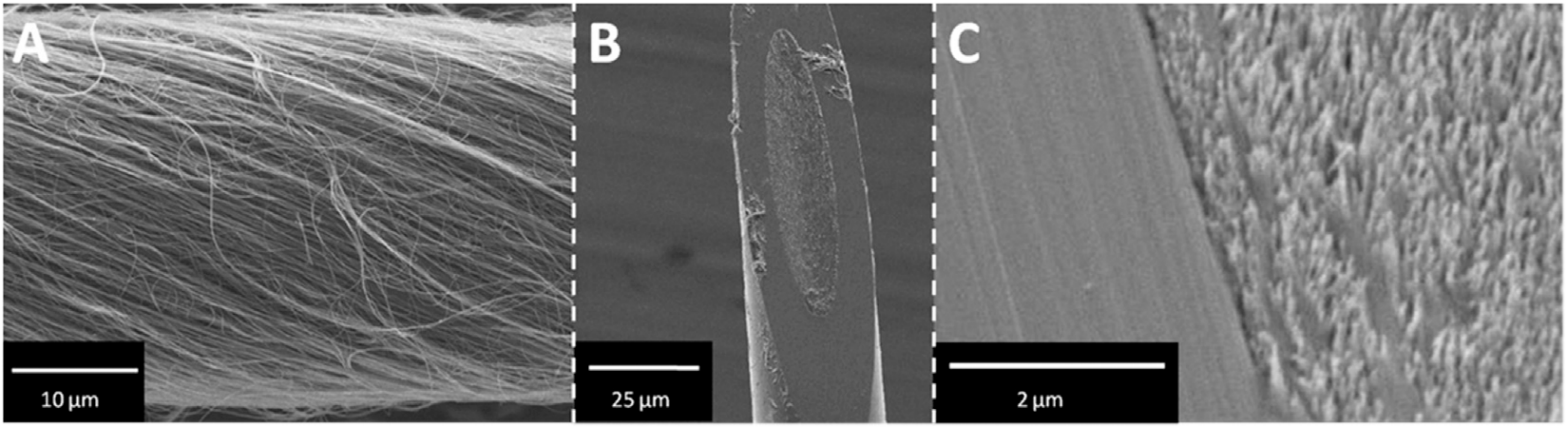
SEM images of CNTY-D electrode. **(A)** Spun MWCNTs form a continuous yarn. **(B)** A single CNTY-D electrode. **(C)** Detailed micrograph of CNT surface and glass seal of the CNTY-D electrode (Schmidt et al., 2013).

These advancements in CNT yarn technology open up new possibilities for studying neurochemistry and behavior by providing more sensitive, selective, faster, and durable measurements. CNT yarn electrodes provide enhanced sensitivity and temporal resolution, allowing researchers to monitor rapid fluctuations in neurotransmitter levels in real-time. This capability is crucial for understanding the dynamic processes of neurotransmitter release and uptake during complex behaviors where neurochemical levels can change within a fraction of a second (Schmidt et al., 2013). The 30 μm diameter carbon nanotube yarn shown in Figure 3A was fabricated into microdisk electrodes with elliptical sensing surfaces (Figure 3B, minor diameter 17.3 ± 0.4 μm), while Figure 3C’s high-magnification SEM imaging revealed individual nanotube ends clearly distinguishable from the glass seal, which is critical for microelectrode sensing applications.

### 2.4 Glassy carbon electrodes

Pyrolyzed photolithographically patterned glassy carbon microelectrodes (GC-MEAs) have emerged as a promising alternative to traditional carbon fiber microelectrodes (CFMEs) due to their superior structural integrity, reproducibility, and scalability for multi-channel sensing. Fabricated through photolithographic patterning and high-temperature pyrolysis of polymeric precursors such as SU-8 or polyimide, GC-MEAs exhibit high electrochemical stability, tunable surface properties, and enhanced mechanical robustness, making them well-suited for chronic implantation (Nam et al., 2022; Siwakoti et al., 2025). Unlike CFMEs, which may degrade or fray over time, GC electrodes maintain consistent performance and can be integrated into high-density arrays for simultaneous multi-site neurochemical detection. Recent advancements in functionalization strategies have further improved their performance, with coatings such as PEDOT and PEDOT:PSS enhancing charge transfer kinetics, reducing impedance, and increasing sensitivity toward neurotransmitters such as dopamine and serotonin (Choi et al., 2023). Additionally, the incorporation of nanocarbon materials, such as graphene and carbon nanotubes, has been shown to significantly improve electrochemical signal strength, neurotransmitter adsorption, and resistance to biofouling. The integration of GC-MEAs with flexible substrates has also enabled the development of minimally invasive, biocompatible neural interfaces that reduce tissue damage and inflammatory responses during long-term implantation (Devi et al., 2021). *In vivo* applications of GC-MEAs have demonstrated stable, real-time neurotransmitter detection and the ability to simultaneously record electrophysiological activity, making them highly valuable for studying neural dynamics and developing bioelectronic interfaces (Castagnola et al., 2024). As research continues to optimize material properties, functional coatings, and integration strategies, GC-MEAs are poised to advance real-time neurochemical monitoring and improve the long-term stability of implantable neural sensors (Siwakoti et al., 2025).

## 3 The impact of microelectrode size on neurochemical sensing

Here, we review the impact of microelectrode design on electrochemical performance, tissue response, and long-term stability. Special attention is given to the trade-offs between electrode size, sensitivity, and biocompatibility, with references to key studies on chronic implantation effects. Microelectrode size plays a crucial role in determining the performance and utility of neurochemical sensors (Purcell et al., 2021). The term “microelectrode” encompasses both single-channel electrodes and multi-channel electrode arrays, each with distinct applications and implications for device design (Xu et al., 2022). Single-channel electrodes typically provide high-resolution recordings of neurochemical activity at a single site, whereas multi-channel electrode arrays enable simultaneous detection at multiple points, enhancing spatial resolution and data acquisition efficiency (Huang et al., 2021; Zestos et al., 2022). The choice between these configurations influences device miniaturization, sensitivity, and tissue compatibility.

Several comprehensive reviews offer insights into the relationship between electrode size, material properties, and biocompatibility. As such, some studies have reported that smaller-diameter electrodes significantly reduce tissue disruption during implantation, enhancing their biocompatibility and enabling precise, localized neurochemical measurements (McNamara et al., 2024). Notably, even though any invasive implantation process elicits some degree of immune response due to tissue disruption, reducing the size of the electrode can minimize tissue damage and attenuate the inflammatory reaction (Iwasa et al., 2020). Studies have shown that smaller-diameter carbon fiber electrodes cause less gliosis and chronic inflammation compared to larger implants, thereby improving long-term biocompatibility and signal stability (Kozai et al., 2015; Fu et al., 2021).

Moreover, reducing the size of microelectrodes improves their spatial resolution and sensitivity. For example, smaller electrodes have been shown to record higher signal amplitudes when in proximity to neurons, a factor critical for high-resolution neurochemical sensing (Moffitt and McIntyre, 2005). The small size facilitates the detection of rapid and localized neurotransmitter dynamics, such as dopamine release, which is central to understanding brain activity and dysfunctions (Huffman and Venton, 2009). Smaller microelectrodes are also more compatible with biological tissues, reducing immune responses and inflammation. For instance, studies show that electrodes with minimized size exhibit better long-term stability *in vivo*, making them suitable for chronic neurochemical monitoring (Yin et al., 2023). Diamond-based microelectrodes, characterized by their corrosion resistance and biocompatibility, are particularly promising for long-term applications (Rusinek et al., 2018). The reduced size of microelectrodes has enabled breakthroughs in neurochemical sensing. For example, carbon fiber microelectrodes are widely used for the detection of dopamine and other neurotransmitters due to their high sensitivity and rapid response time (He et al., 2022). Moreover, advances in microelectrode design have facilitated the development of multi-functional arrays capable of simultaneously recording electrophysiological and neurochemical signals (Johnson et al., 2006).

Recent advances in microfabrication techniques, including laser etching, photolithography, and electrophoretic deposition, have enabled the development of ultra-thin and highly specialized microelectrodes. These methods allow for precise control of electrode dimensions and surface properties, leading to improved signal-to-noise ratios and sensitivity (Tan et al., 2018). Furthermore, novel materials like graphene and carbon nanotubes are being incorporated to enhance the electrochemical performance of these microelectrodes, offering superior electron transfer kinetics and surface fouling resistance (Li et al., 2022).

## 4 Stability, biocompatibility, and performance

The stability and biocompatibility of carbon microelectrodes are critical factors that enable their long-term functionality in neurochemical sensing. These properties are essential for ensuring consistent performance during acute and chronic studies, particularly for *in vivo* applications where immune responses and tissue interactions play a significant role. Surface functionalization has been a pivotal approach to enhance the performance of carbon microelectrodes. The application of nanomaterials such as graphene and carbon nanotubes (CNTs) improves electrode conductivity and reduces biofouling, thereby maintaining high sensitivity over extended periods (Fang et al., 2015). For instance, CNTs not only increase the electrochemical surface area but also improve the selectivity and sensitivity of electrodes by creating active sites for specific analyte interactions (Liu et al., 2006).

Innovative fabrication techniques have further enhanced electrode stability. As such, the incorporation of 3D pyrolytic carbon microelectrodes provides increased surface area and superior electrochemical properties, making them more durable and effective for neurochemical sensing (Hemanth et al., 2017). Moreover, the use of hybrid materials, such as graphene-coated carbon microelectrodes, has demonstrated higher sensitivity and reduced noise levels, allowing for accurate neurotransmitter detection in complex environments (Kshirsagar et al., 2018). A significant factor influencing electrode stability is tissue response, which can lead to inflammation, gliosis, and encapsulation, ultimately affecting analyte diffusion and electrochemical performance. This issue has been addressed by developing micro-invasive probes (µIPs), which dramatically reduced tissue response and enabled stable, chronic subsecond monitoring of dopamine *in vivo* (Schwerdt et al., 2018). Their study demonstrated that reducing electrode diameter to cellular scale (<10 µm) minimized chronic tissue disruption, resulting in significantly lower gliosis and immune activation compared to conventional CFMEs. Furthermore, the use of parylene-C insulation and controlled etching techniques ensured stable electrode function for over a year, a significant improvement over standard CFMEs, which typically exhibit signal degradation within weeks to months.

## 5 Fabrication techniques for improved durability and cost-effectiveness

Advancements in fabrication techniques have significantly improved the durability and cost-effectiveness of carbon microelectrodes, making them accessible for diverse applications, from neuroscience to microfluidics. Innovations in methods such as chemical vapor deposition (CVD), 3D printing, and laser direct writing have contributed to the production of durable and affordable electrodes. CVD is a widely used method for fabricating high-performance carbon microelectrodes. As such, the integration of CNTs into microelectrodes using CVD has demonstrated exceptional durability, mechanical strength, and low electrical resistance, making it suitable for long-term applications in neural sensing (Mishra et al., 2018). Additionally, this process facilitates the development of high-aspect-ratio carbon microelectrodes, essential for minimizing tissue damage during implantation (Chen et al., 2018).

Recent advancements in 3D printing have enabled the fabrication of cost-effective microelectrode arrays. By using customizable 3D-printed molds and biocompatible polyimide resins, researchers have developed batch production methods that significantly lower production costs while maintaining high performance. These electrodes have shown excellent sensitivity in detecting neurotransmitters and can be produced in large quantities, making them ideal for clinical and research use (Trikantzopoulos et al., 2016). Notably, inkjet-printed carbon microelectrodes can be produced for mere cents, with rapid prototyping capabilities allowing for quick development (Schnitker et al., 2018).

Laser direct writing (LDW) is a versatile and cost-effective approach for fabricating carbon microelectrodes. LDW utilizes CO_2_ lasers to convert polyimide into porous carbon, achieving high-quality laser-induced graphene (LIG) structures with low sheet resistance (71 ± 15 Ω sq.) (Murray et al., 2022). The process involves carbonization followed by graphitization, enhancing conductivity from 56.1 S m^−1^ to 146.7 S m^−1^ (Biswas et al., 2020). These electrodes exhibit excellent flexibility, high electrical conductivity, and durability, making them suitable for applications such as electrochemical sensing. Surface treatments like plasma etching further enhance electrode performance by improving contact interfaces and increasing capacitance (Murray et al., 2022). Inkjet printing offers a low-cost alternative for producing carbon microelectrodes. This method utilizes carbon nanotube-based inks to create microelectrode patterns with high spatial resolution (Schnitker et al., 2018). The simplicity of this approach makes it accessible to laboratories without advanced lithography facilities, reducing barriers to adoption for research and clinical applications (Cisotto et al., 2019; da Costa and Choi, 2020). Other studies have demonstrated the production of inkjet-printed single-walled carbon nanotube (SWCNT) thin films demonstrating excellent mechanical properties and conductivity, maintaining performance under strain (Kim et al., 2014).

The incorporation of nanomaterials such as graphene and CNTs into microelectrodes has revolutionized their fabrication. Graphene and CNTs exhibit exceptional mechanical strength and flexibility, which are crucial for applications in soft tissue environments. For instance, CNTs provide a one-dimensional conductive path that maintains electrical integrity under strain, as demonstrated in flexible transparent conductive films (Lee and Jeong, 2013). The incorporation of CNTs into microelectrode arrays has shown a reduction in overall impedance, enhancing signal transmission and stability, which is vital for long-term neural applications (Vafaiee et al., 2020). CNTs have been functionalized to improve hydrophilicity, significantly reducing protein absorption and inflammatory responses, which are common issues in neural electrodes. This functionalization leads to lower impedance and better performance over time (Alvarez and Ruhunage, 2023). The combination of CNTs and manganese dioxide (MnO_2_) in 3D carbon microelectrodes has demonstrated remarkable stability and performance in electrochemical applications, further emphasizing the benefits of nanomaterial integration (Jiang et al., 2014; Biswas et al., 2024).

## 6 Emerging materials for carbon microelectrodes

Emerging materials, including doped carbon nanomaterials and hybrid composites, are paving the way for the next-generation of carbon microelectrodes. These materials provide enhanced electrochemical properties, such as improved sensitivity, reduced noise, and better biocompatibility, enabling the detection of low-abundance neurotransmitters in complex biological systems.

### 6.1 Doped carbon nanomaterials

Nitrogen-doped carbon materials have gained significant attention due to their ability to improve electron transfer kinetics, enhance conductivity, and provide additional active sites for electrochemical reactions (Talukder et al., 2021; Yuan, 2022). Techniques like chemical vapor deposition (CVD) are employed to create nitrogen-doped graphene nanosheets and carbon nanotubes, which exhibit enhanced mechanical properties (Steinmetz et al., 2020). The use of nitrogen-rich precursors during carbonization leads to significant improvements in the structural integrity and electrochemical characteristics of the resulting materials (Yuan, 2022).

**FIGURE 4:**
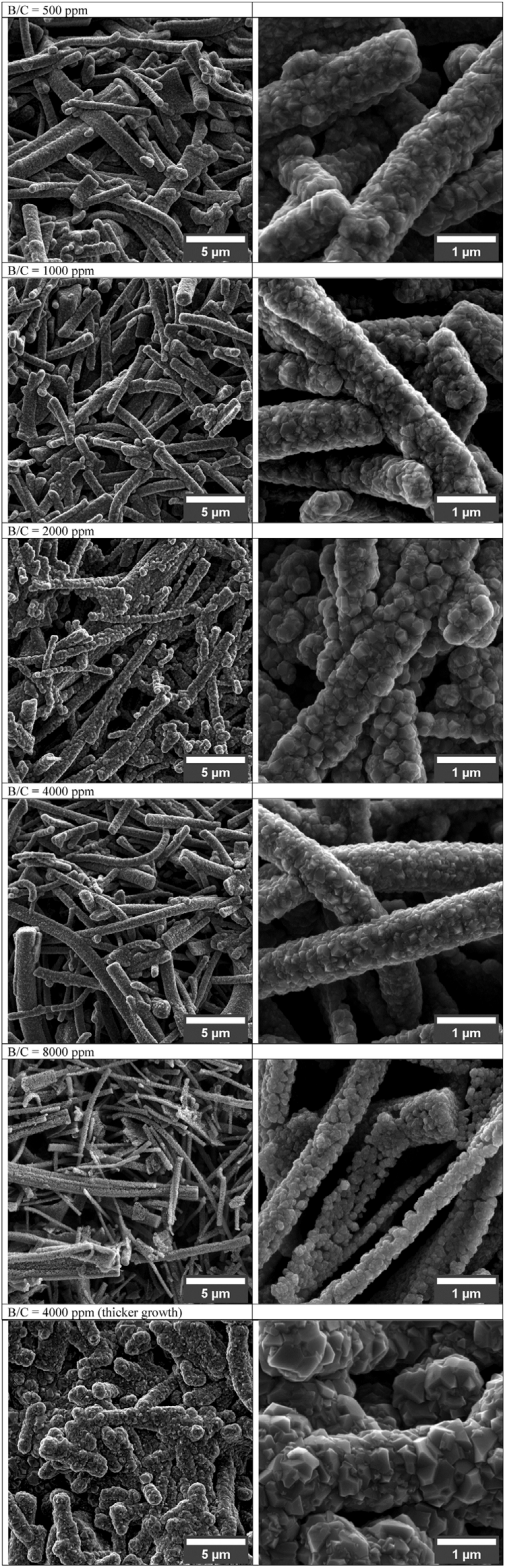
SEM micrographs of porous BDD layers grown with increasing B/C ratio (500 up to 8,000 ppm) (Baluchova et al., 2019).

Boron-doped diamond (BDD) is another widely explored material in electrochemical sensing due to its unique properties, such as a wide potential window, relatively low background noise, and chemical inertness (Luong et al., 2009; Gong et al., 2023). Boron doping introduces p-type semiconducting behavior in diamond, enhancing its electrochemical activity (Einaga, 2022). BDD electrodes have been successfully utilized in fast-scan cyclic voltammetry (FSCV) for dopamine detection, showing a wide potential window and low noise. However, the FSCV signal for dopamine remains small, even at high concentrations such as 20 μM (Granger et al., 2000). BDD also demonstrates excellent antifouling properties compared to carbon fiber microelectrodes (CFMEs). Studies have shown that BDD exhibits reduced fouling during dopamine measurements, making it highly suitable for human studies (Yang et al., 2020). Furthermore, modifications like porous structures in BDD films significantly enhance their electroactive area and sensitivity for dopamine detection (Baluchova et al., 2019). Figure 4 demonstrates how boron-doped diamond (BDD) films with varying B/C ratios affect electrochemical detection capabilities, showing that planar layers maintain polycrystalline structure regardless of doping, while porous BDD films completely cover SiO_2_ templates only up to 4,000 ppm B/C ratio before coverage deteriorates at 8000ppm due to reduced growth quality; additionally, extended growth time at 4,000 ppm results in complete template coverage but with diminished porosity. Boron-doped CNTs are another important development in electrochemical sensing. Doping increases the D-band to G-band peak intensity (D/G) ratio of CNTs, improves their electrocatalytic activity, and enhances peak currents for specific analytes (Liao et al., 2023). However, excessive boron doping can compromise the conductivity and overall performance of CNTs (Salazar et al., 2012).

### 6.2 Hybrid composites for superior properties

Hybrid composites that combine conductive polymers with carbon nanofibers or graphene have shown exceptional promise in enhancing the performance of microelectrodes. These materials reduce impedance, improve charge transfer capacity, and enhance neurotransmitter detection capabilities. For instance, PEDOT-carbon nanofiber composites exhibit improved biocompatibility and enhanced signal-to-noise ratios, making them particularly effective for long-term neural recordings (Saunier et al., 2020b). Studies have demonstrated that optimized PEDOT-based coatings can significantly improve neurotransmitter sensitivity while maintaining low impedance. This makes them particularly effective for detecting dopamine and serotonin at low concentrations (Saunier et al., 2020a). However, excessive deposition of polymer layers can hinder fast-scan cyclic voltammetry (FSCV) applications by introducing capacitive charging currents. This emphasizes the need for precise fabrication control to maintain both high sensitivity and compatibility with neurochemical sensing methods.

Additionally, hybrid structures incorporating carbon nanotubes (CNTs) and boron-doped diamond (BDD) electrodes have demonstrated enhanced electrocatalytic activity. The synergistic effects of these materials offer increased stability and a broader potential window, which is advantageous for neurochemical analysis. Studies have demonstrated that, nickel-encapsulated CNTs grown on BDD substrates exhibit improved electrochemical performance due to the synergistic effects of the two materials which offers increased sensitivity and a broader linear range for applications such as glucose sensing (Long et al., 2018). These advancements highlight the importance of integrating multi-functional materials to address both electrical and biochemical challenges in neurochemical sensing.

### 6.3 Biocompatible and functionalized surfaces

The integration of bioactive surfaces with doped carbon materials addresses challenges such as biofouling and tissue reactivity. Functionalized carbon electrodes with bio-compatible polymers, such as poly (thiophene)-based coatings, have shown potential in enhancing long-term neural recordings while minimizing immune responses (Kozai et al., 2012). Studies indicate that these coatings maintain lower biofouling levels for extended periods, thereby improving the overall performance of the electrodes. As such, carbon nanotubes (CNTs) coated with biocompatible polymers have demonstrated reduced impedance and increased hydrophilicity, which help limit inflammatory reactions and enhance signal consistency over time (Srivastava, 2017; Ruhunage et al., 2022).

Recent studies have explored the use of peptide-functionalized electrodes that promote neuronal adhesion and reduce inflammatory glial responses (Cui, 2010). These modifications improve electrode longevity and reduce signal degradation over time. Moreover, surface engineering techniques, including plasma treatments and nano-structuring, have been employed to enhance the biocompatibility of carbon microelectrodes (Ates and Sarac, 2009; Cui, 2010). Future research should focus on developing adaptive coatings that dynamically respond to the neurochemical environment, ensuring sustained functionality and minimal tissue disruption. By integrating advanced material science strategies, microelectrodes can achieve improved stability, sensitivity, and long-term *in vivo* performance.

#### 6.3.1 Voltammetric techniques used with carbon microelectrodes

Electrochemical methods have been widely used for the detection of neurotransmitters due to their high temporal resolution, sensitivity, and ability to detect neurotransmitters in real-time. Among these, voltammetric techniques such as Fast-Scan Cyclic Voltammetry (FSCV) have been extensively utilized with carbon microelectrodes (CMEs) for neurotransmitter sensing (Chuntib et al., 2017; Venton and Cao, 2020).

#### 6.3.2 Fast-scan cyclic voltammetry (FSCV)

FSCV is a widely used electrochemical technique for detecting rapid changes in neurotransmitter concentrations, particularly catecholamines such as dopamine, norepinephrine, and serotonin (Rafi and Zestos, 2021). FSCV applies a triangular waveform potential at a high scan rate (typically 400 V/s) to a carbon fiber microelectrode, rapidly cycling between a holding and switching potential (Puthongkham and Venton, 2020). This method allows for sub-second detection of neurotransmitter fluctuations *in vivo* with high spatial and temporal resolution (Roberts and Sombers, 2017).

The main advantage of FSCV is its ability to detect neurotransmitters in real time with minimal sensor drift (Venton and Cao, 2020). However, it also generates a high background current due to the rapid charging and discharging of the electrode-electrolyte interface, which must be subtracted to extract meaningful signals (Dorta Quinones, 2014). This technique has been successfully used in animal models to study neurochemical dynamics in regions such as the striatum and prefrontal cortex (Grinevich et al., 2022).

#### 6.3.3 Operating mechanism

**SCHEME 1:**
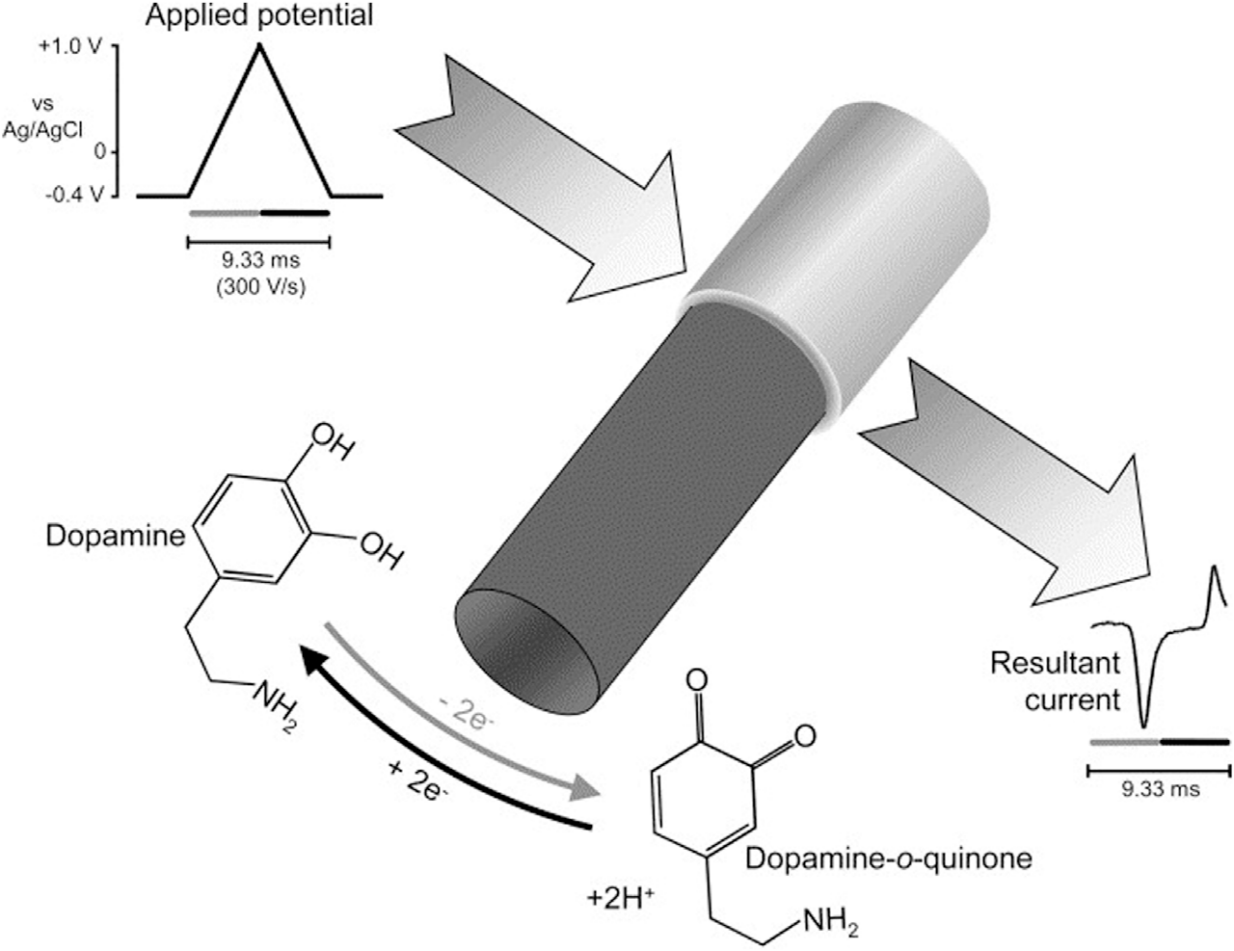
Oxidation of dopamine in FSCV (Phillips et. al 2003; Niyonambaza et al., 2019).

FSCV operates by applying a triangular voltage waveform at high scan rates, typically between 400 V/s and 1000 V/s, to a carbon fiber microelectrode (CFME). The waveform cycles between a holding potential, such as −0.4 V (vs. Ag/AgCl), and an oxidation potential, which varies depending on the target analyte (Puthongkham and Venton, 2020). For instance, dopamine is oxidized at approximately +0.6 V, where it loses electrons and is converted into dopamine-o-quinone. On the reverse scan, the applied potential returns to the holding potential, allowing the reduced form of dopamine to be regenerated. The resulting oxidation and reduction currents are recorded as a cyclic voltammogram, where peak current intensity is proportional to neurotransmitter concentration. Scheme 1 illustrates the oxidation of dopamine in FSCV (Niyonambaza et al., 2019). Since FSCV operates at high frequencies, often at intervals of 10–100 milliseconds, it enables researchers to track rapid neurotransmitter changes in living brain tissue, an essential capability for understanding synaptic activity and neurochemical signaling (Wu et al., 2018).

Carbon fiber microelectrodes (CFMEs) have become the gold standard for FSCV due to their small diameter (5–10 µm), which minimizes tissue damage upon implantation and allows for high spatial resolution in neurochemical measurements (Ostertag and Ross, 2023). These electrodes exhibit excellent conductivity, supporting fast electron transfer kinetics essential for high-speed neurotransmitter detection. Their low background noise and ability to be functionalized with materials such as Nafion and PEDOT further enhance their selectivity and sensitivity (Taylor et al., 2017). Additionally, CFMEs possess a fast response time, which is critical for monitoring the rapid release and uptake of neurotransmitters. Due to their high biocompatibility and mechanical stability, CFMEs are widely used in both acute and chronic neurochemical studies.

The detection mechanism in FSCV relies on the electrochemical oxidation and reduction of neurotransmitters, with each neurotransmitter exhibiting a distinct redox potential profile. Dopamine, for instance, undergoes a two-electron oxidation at approximately +0.6 V to form dopamine-o-quinone, followed by a reduction back to dopamine during the reverse scan. Similarly, serotonin (5-HT) and norepinephrine (NE) display oxidation peaks at approximately +0.6 V and +0.8 V, respectively, allowing FSCV to differentiate among neurotransmitters based on their unique electrochemical signatures (Ma et al., 2012; Dunham and Venton, 2020). Table 1 and Table 2 illustrate various neurotransmitters along with their detection potentials The detection process follows the general reaction: Neurotransmitter→ (Oxidized Form) +e^−^ + H^+^. For example, dopamine undergoes a two-electron oxidation to dopamine-o-quinone as illustrated in Scheme 1.

**TABLE 1:** Comparison of carbon electrode materials for neurotransmitter detection.

Electrode material	Size diameter	Selectivity	Sensitivity (LOD)	Temporal resolution	Stability	Fouling resistance	Cost	References
CFME	5–30 μm	Moderate	0.86 ± 0.19 μM	High (ms)	Moderate	Low to moderate	Low	Wang et al. (2019)
CNT Yarn	5–50 μm	High	13.4 ± 1.7 nM to 20.8 ± 1.3 nM	High (ms)	High	High	Moderate	Schmidt et al. (2013)
BDD (Boron-Doped Diamond)	10–50 μm	Very high	2 × 10^−7^ M	Moderate (tens of ms)	Very high	Very high	High	Luong et al. (2009), Baluchova et al. (2019)
MWCNT-Composite	10–100 μm	High	0.2 μM–0.01 μM	High (ms)	High	Moderate to high	Moderate	Wang et al. (2015)

**TABLE 2:** Oxidation potentials of various neurotransmitters.

Neurotransmitter	Oxidation peak (V vs. Ag/AgCl)	Reduction peak (V vs. Ag/AgCl)	Detection characteristics	References
Dopamine (DA)	+0.6 V	−0.2 V	High selectivity, fast adsorption kinetics	Niyonambaza et al. (2019)
Serotonin (5-HT)	+0.6 V	−0.2 V	Similar to DA, but lower sensitivity	Dunham and Venton (2020)
Norepinephrine (NE)	+0.8 V	−0.2 V	Oxidation occurs at higher potentials than DA	Ma et al. (2012)
Epinephrine (EP)	+0.9 V	−0.3 V	Stronger adsorption to CFME surfaces	Ma et al. (2012)

#### 6.3.4 Challenges and optimization in FSCV electrochemical detection

While FSCV provides unparalleled temporal resolution for neurotransmitter detection, several challenges must be addressed to ensure accurate and reliable measurements. One major limitation is the presence of background charging current, which results from the rapid potential sweeps. This background current can be significantly larger than the faradaic current generated by neurotransmitter oxidation, necessitating precise subtraction methods to isolate relevant signals (Lucio Boschen et al., 2021). Another challenge is electrode fouling, which occurs when oxidation byproducts adsorb onto the electrode surface, gradually reducing sensitivity. Strategies such as waveform optimization and surface functionalization with antifouling coatings have been developed to mitigate this issue (Taylor et al., 2017; Jang et al., 2024). Furthermore, while FSCV is highly sensitive to catecholamines, it lacks absolute specificity, as neurotransmitters with similar oxidation potentials can produce overlapping voltammetric signatures. To enhance selectivity, researchers have employed chemically selective coatings, machine learning-assisted signal processing, and modified waveforms tailored for specific analytes (Venton and Cao, 2020).

Despite these challenges, FSCV with CFMEs remains one of the most powerful techniques for *in vivo* neurotransmitter sensing. Its ability to provide real-time measurements with sub-second resolution has revolutionized the study of neurochemical dynamics in both healthy and diseased brain states. Future advancements in electrode materials, waveform design, and computational signal analysis are expected to further refine FSCV technology, improving its selectivity, longevity, and adaptability for clinical applications (Avula et al., 2023; Perillo et al., 2025). As research continues to optimize this technique, FSCV will play an increasingly vital role in understanding the neurochemical basis of behavior, learning, and neurological disorders.

## 7 Clinical applications in neurological disorders

FSCV has revolutionized the field of neurochemical monitoring by facilitating real-time and high-resolution detection of neurochemicals, providing valuable insights into the dynamic changes of neurotransmitters in the brain. By using a rapidly changing electrical potential at a microelectrode, FSCV enables the measurement of neurotransmitter concentrations with excellent temporal resolution (Puthongkham and Venton, 2020). FSCV coupled with CFMEs has emerged as a powerful technique for monitoring neurochemicals, such as dopamine, and studying their role in neurological disorders such as Parkinson’s Disease (Smith, 2021). This method has been instrumental in investigating the release, reuptake, and signaling pathways of neurotransmitters such as dopamine, providing critical insights into its involvement in complex behaviors, learning, and neurodegenerative diseases. The sensitive and selective measurement of neurotransmitters is also crucially important for studying other brain disorders such as depression and addiction among others. Carbon microelectrodes have transitioned from laboratory research tools to promising clinical applications, particularly in diagnosing and monitoring neurological disorders.

### 7.1 Parkinson’s disease

Parkinson’s Disease is a neurodegenerative disorder caused by the death of dopaminergic neurons that induces tremors, stiffness, and bradykinesia (Shah et al., 2020). The use of FSCV with CFMEs is useful for detecting dopamine, serotonin, adenosine, and histamine with high spatiotemporal resolution. This monitoring helps researchers understand Parkinson’s disease’s neurochemical changes, improving treatment and patient outcomes (Xu et al., 2023). Studies have shown the effectiveness of FSCV research with CFMEs where Parkinsonian patients’ striatum serotonin signaling was examined, specifically serotonin concentrations changed with sequential investment game outcomes and decisions (Moran et al., 2018). Negative reward prediction mistakes increased serotonin levels, but counterfactual losses decreased them. These data corroborate theoretical hypotheses that serotonin opposes dopamine signaling (Moran et al., 2018).

Other researchers have adopted FSCV to measure dopamine release in the human striatum during Parkinson’s disease treatment (Kishida et al., 2016). The deep brain stimulation (DBS) electrode’s effectiveness and carbon fiber probe remained intact during insertion. This study provides a better understanding pertaining to the treatment of Parkinson’s disease by revealing dopamine neuron activity in depleted systems. Koehne et al. used carbon nanofiber (CNF) electrode arrays with the Wireless Instantaneous Neurotransmitter Concentration Sensor System (WINCS) to measure dopamine in patients with Parkinson’s Disease. CNF arrays detected dopamine similarly to CFMs (Koehne et al., 2011). This shows that CNF arrays could replace carbon electrodes for neurochemical monitoring, perhaps helping treat Parkinson’s Disease. CNF arrays with FSCV help researchers study dopamine dynamics and design new Parkinson’s Disease treatments. Parkinson’s Disease diagnostics and treatment could potentially be enhanced through the use of these biosensors (Koehne et al., 2011).

Other studies developed durable, synthetic boron-doped diamond-based (BDD) electrodes to measure human neurochemical release (Bennet et al., 2016). Diamond electrodes surpassed carbon fiber electrodes by more than two orders of magnitude in physical robustness (strength and rigidity) and *in vitro* lifetime. These diamond electrodes targeted the thalamus in humans receiving deep brain stimulation (DBS) for tremor for the first time. BDD electrodes are not expected to be conductive because they are sp^3^ hybridized in contrast to sp^2^ hybridized CFMEs but undergo p-doping (positive doping) with boron to increase conductivity. Diamond electrodes recorded adenosine release, a neurochemical that modulates tremors, with sensitivity comparable to carbon fiber electrodes. This electrode technology development could improve the precision and efficacy of deep brain stimulation for Parkinson’s disease (Bennet et al., 2016). Another study coupled FSCV to the Wireless Instantaneous Neurotransmitter Concentration Sensing (WINCS) device to monitor histamine through waveform optimization. The WINCS method reliably detected histamine release from electrical stimulation at the tuberomammillary nucleus in rat brain slices. The WINCS system was shown to monitor histamine levels and help us understand its involvement in Parkinson’s Disease and other physiological and pathological processes, which could improve treatment (Chang et al., 2012).

### 7.2 Depression and neuropsychiatric disorders

Depression is a mental health disorder linked to the depletion of extracellular serotonin levels in the brain (Albert et al., 2012). Serotonin is an important chemical that helps control mood and its imbalance can lead to depressive symptoms such as low self-worth and feelings of guilt (Tahiri et al., 2024). Electrochemical sensors, particularly those utilizing carbon nanotubes (CNTs), have been employed to monitor serotonin levels, providing insights into the neurochemical basis of depression and aiding in the development of targeted therapies (Jacobs et al., 2011). One study developed an electrochemical sensor based on a Cu_2_O-CNT core decorated with platinum nanoparticles. This sensor exhibited a low detection limit of 3 nM for serotonin, showcasing its potential for early disease diagnostics by providing a sensitive and low-cost platform for serotonin detection (Ashraf et al., 2021). Others developed a polymelamine modified Edge Plane Pyrolytic Graphite Sensors (EPPGS) for the determination of serotonin. This sensor exhibited excellent electrocatalytic activity towards serotonin oxidation with a detection limit of 30 nM, and was effective in determining serotonin levels in human blood serum and urine (Gupta and Goyal, 2014).

Furthermore, advanced electrochemical methods have been developed using FSCV to explore the link between inflammation and depression (Hashemi et al., 2011). They developed techniques to simultaneously detect histamine and serotonin *in vivo* in mammals, employing the Jackson (serotonin) waveform with Nafion-coated electrodes (Hashemi et al., 2011). Their research revealed that serotonin and histamine levels fluctuate with prolonged stress and escitalopram treatment. Another study observed an increase in histamine and a decrease in serotonin in a stressed mice (Denno et al., 2015).

### 7.3 Drug abuse and dopamine detection

Drug abuse, including substances such as cocaine, amphetamine, bath salts, and opioids, significantly affects dopamine signaling pathways by increasing extracellular dopamine. Psychostimulants such as cocaine and amphetamine serve as substrate releasers or blockers of the dopamine transporter (DAT), serotonin transporter (SERT), and the norepinephrine transporter (NET), respectively (Plaisance et al., 2024). They increase extracellular levels of dopamine and other monoamine neurotransmitters, which can cause addiction, craving, tachycardia, and immense feelings of reward (Mani et al., 2020; Wise and Jordan, 2021). The capability of monitoring rapid changes in dopamine levels is crucial for understanding the neurochemical effects of these drugs.

Carbon-based electrodes, due to their high sensitivity and fast response times, are ideal for detecting dopamine fluctuations in response to drug exposure. Studies have demonstrated the utility of these sensors in tracking the acute and chronic effects of drug abuse on neurotransmitter dynamics (Song et al., 2023). The brain’s reward system’s dynamic cellular and molecular responses must be studied in real time at a sub-second level to understand drug addiction’s neurobiology. Numerous electrophysiological and electrochemical investigations have examined cell firing patterns and fast dopamine signaling during key drug-seeking and drug-taking behaviors. These studies show how these signals are associative and tied to addiction (Carelli and Wightman, 2004). By studying these spontaneous reactions, we can learn more about the mechanisms of drugs abuse and action in the brain behind addiction and create more effective treatments.

**FIGURE 5:**
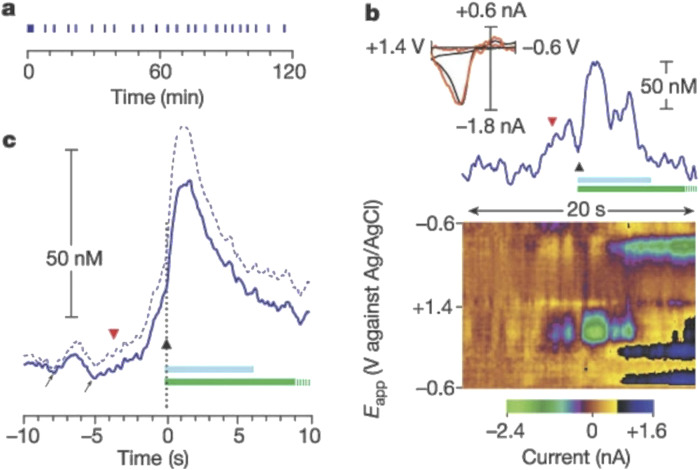
Rapid increase in extracellular dopamine in the nucleus accumbens relative to the lever-press response for cocaine. **(a)** Individual lever-press responses (vertical lines) for a representative rat are shown against time in the session. **(b)** The red, inverted triangle denotes the final lever approach before a lever press, represented by the black triangle, during this session. The light-blue bar represents cocaine infusion (0.33 mg, 6 s); the green bar represents the audiovisual stimulus (20 s), which is truncated at 10 s. The top part shows the time course of dopamine concentration change. Inset, the cyclic voltammogram obtained at the maximal dopamine change (red line) is superimposed on a current-normalized one obtained during electrical stimulation (black line). **(c)** The solid blue line is the mean dopamine change across all animals (n = 6) around the lever press, and the dashed blue line denoted the mean +standard error. Increases in dopamine before the lever press are highlighted by the arrows. Reproduced with permission from (Phillips et al., 2003).

When used *in vivo*, CFMEs are useful for researching neurobiological mechanisms and pharmacological effects, including addictive medications. CFMEs can now capture natural dopamine release during behavioral trials due to advances in sensitivity and equipment. This feature allows researchers to study the complex relationship between brain activity, dopamine signaling, and behavior (Huffman and Venton, 2009). Drug-induced dopamine release has been studied using CFMEs. Cocaine, ethanol, and nicotine have been shown to affect dopamine dynamics without electrical stimulation. Cocaine increases quick dopamine transients, which are intense spikes in dopamine release, and gradually raises basal dopamine levels as observed by Cheer et al. (Cheer et al., 2004). Also, this work showed that intravenous cocaine, ethanol, and nicotine boost brain dopamine levels immediately. These findings illuminate the complex neurochemical systems that respond to addictive compounds and help us comprehend their immediate impact on brain dopamine signaling. Moreover, investigators also found that sub-second dopamine release measured by voltammetry promoted cocaine seeking in animals that were trained to lever press for cocaine (Phillips et al., 2003) (Figure 5). This groundbreaking study demonstrated how cue-induced dopamine release could be measured using carbon fiber microelectrodes (CFMEs) and fast-scan cyclic voltammetry (FSCV) in trained mice before their cocaine self-administration.

CNT electrodes enhance sensitivity and reduce fouling, making them suitable for *in vivo* measurements of neurotransmitter changes induced by opioids (Swamy and Venton, 2007). An electrochemical aptasensor using single-walled carbon nanotubes (SWCNTs), gold electrodes, and complementary strands of aptamer has been developed for sensitive and selective cocaine detection. This sensor demonstrated a limit of detection as low as 105 pM, highlighting its high sensitivity and applicability for real sample analysis (Taghdisi et al., 2015). The method of using CFMEs with FSCV has been effective in detecting dopamine changes in response to psychostimulants like cocaine and amphetamine, providing insights into the dynamic neurochemical environment and complex behaviors influenced by these drugs (Zestos et al., 2022). Corona phase molecular recognition (CoPhMoRe) on fluorescent SWCNT sensors has been employed to detect neurotransmitters like dopamine with high selectivity. These sensors exhibit a fluorescence response to dopamine, enabling real-time monitoring of neurotransmitter levels, which is crucial for studying the effects of drugs such as bath salts on dopamine signaling (Kruss et al., 2014). Carbon-doped hexagonal boron nitrogen (C-hBN) sensors have shown enhanced electrocatalytic activity towards dopamine redox reactions. These sensors offer high sensitivity and low detection limits, making them effective for detecting dopamine changes in response to various drugs, including amphetamines (Ouyang et al., 2021).

## 8 Conclusion and future directions

The advancements in carbon microelectrode (CME) technology have significantly expanded the horizons of neurochemical sensing. By leveraging cutting-edge fabrication techniques, such as chemical vapor deposition, 3D printing, and laser direct writing, CMEs have been utilized to achieve new levels of sensitivity, durability, and cost-effectiveness. The integration of nanomaterials, including carbon nanotubes and graphene, has further enhanced their performance, enabling precise, real-time detection of neurotransmitters and overcoming challenges like biofouling and poor selectivity. The development of polymer-modified electrodes and hybrid composites has demonstrated substantial promise in reducing interference and improving the electrochemical properties of CMEs for use in complex biological environments. These advancements have not only supported breakthroughs in basic neuroscience research, but also hold tremendous potential for clinical applications, particularly in diagnosing and treating neurological and psychiatric disorders such as Parkinson’s disease, depression, and epilepsy. Despite these significant strides, challenges remain in ensuring the scalability and long-term stability of CM Es, particularly for *in vivo* applications.

The future of carbon microelectrode technology lies in the integration of these advanced materials with cutting-edge signal processing techniques. As such, some studies have reported that combining electrochemical sensors with CMOS technology allows for miniaturization and improved signal-to-noise ratios (Gill and Ghoreishizadeh, 2023). Additionally, techniques such as laser and plasma treatments can optimize surface structures, increasing active sites for neurotransmitter adsorption (Thangavelu and Duraisamy, 2024). To enhance selectivity and sensitivity, advanced materials with edge-plane sites and functional groups significantly improve dopamine detection. For instance, nitrogen-doped carbon nanopolyhedra can effectively separate dopamine from ascorbic acid interference (Chen et al., 2024). Coatings like PEDOT and Nafion enhance selectivity by rejecting interfering substances, crucial for accurate neurotransmitter monitoring (Rajarathinam et al., 2023). For *in vivo* applications, carbon nanomaterials exhibit inherent resistance to fouling, and polymer coatings can further enhance this property, ensuring reliable *in vivo* measurements (Liu et al., 2023). Furthermore, the use of 3D printing to fabricate hybrid microelectrodes with complex geometries may open new possibilities for personalized neural interfaces.

Future research should focus on optimizing the biocompatibility of materials and integrating emerging technologies, such as microfluidics and machine learning, to enhance the selectivity and functionality of these sensors. The integration of biosensors with optogenetics further adds future promise for these measurements. By addressing these challenges, CMEs can play a pivotal role in revolutionizing personalized medicine and advancing our understanding of the neurochemical underpinnings of behavior and disease. This manuscript highlights the transformative potential of CMEs in both research and clinical domains, emphasizing their versatility and critical importance in understanding and addressing the complexities of neurochemical signaling. The continued evolution of this technology will undoubtedly pave the way for innovative diagnostic tools and therapeutic strategies.
